# Supplementary material for: Sexual Function After Laser Therapy for Penile Cancer: A Systematic Review
Source: Cancers (Basel). 2025 Nov 22;17(23):3737. doi: 10.3390/cancers17233737 (PMC12691219; doi:10.3390/cancers17233737)
Supplement: Supplementary file 1 [file cancers-17-03737-s001.zip › cancers-3940951-supplementary.pdf]

Supplementary Materials:

Supplementary Table S1. Summary of Findings After CO_2 Laser Therapy.

Category	Skeppner Study (2008)[10]	Bandieramonte Study (2008)[11]	Conejo-Mir Study (2005)[12]
Laser Type	CO_2	CO_2	CO_2 laser
Study Title	Treatment-Seeking, Aspects of Sexual Activity and Life Satisfaction in Men with Laser-Treated Penile Carcinoma	Peniscopically Controlled CO_2 Laser Excision for Conservative Treatment of In Situ and T1 Penile Carcinoma: Report on 224 Patients	Carbon Dioxide Laser Treatment of Erythroplasia of Queyrat: A Revisited Treatment to This Condition
Study Design	Retrospective Interview Study	Retrospective Study	Retrospective Study
Sample Size	46 patients (out of 67 treated)	224 patients	8 patients
Age Range/Mean Age	Range: 34-90 years, Median: 63.5 years	Range: 20-83 years, Median: 57 years	Mean: 64 years (Range: 44-70)
Cancer Stage	Tis (13 patients), T1 (14 patients), T2 (19 patients)	Tis (47.3%), T1 (52.7%)	Carcinoma in situ (Erythroplasia of Queyrat) all cases
Post-treatment Pain or Discomfort	N/A	Minimal; pain resolved within 3 days post-op	Minimal; full re-epithelialization within 14-28 days
Social & Relationship Impact	37 of 46 had a steady partner. 31 had the same partner as before treatment. 5 had changed partners.	N/A	N/A
Main Findings	Laser treatment preserves function and quality of life; patients maintain sexual activity and life satisfaction	CO_2 laser excision under peniscopic control provides excellent oncological and functional outcomes for early-stage penile carcinoma	CO_2 laser is an effective, safe, and cosmetically favorable treatment for Erythroplasia of Queyrat
Time to Sexual Activity Resumption	Most resumed within weeks to months. 6% within weeks. 59% within months.	N/A	N/A
Tissue Preservation Success	Complete penile preservation in treated patients	High; complete organ preservation in all but 9 cases requiring amputation	Complete organ preservation in all patients
Satisfaction with Urinary Function	N/A	N/A	No urinary dysfunction observed
Authors	Elisabet Skeppner, Torgny Windahl, Swen-Olof Andersson, Kerstin S. Fugl-Meyer	Gaetano Bandieramonte, Maurizio Colecchia, Luigi Mariani, Salvatore Lo Vullo, Giorgio Pizzocaro, Luigi Piva, Nicola Nicolai, Roberto Salvioni, Valentina Lezzi, Bernardina Stefanon, Giuseppe De Palo	J.S. Conejo-Mir, M.A. Muñoz, M. Linares, L. Rodríguez, A. Serrano
Additional Treatments	Lymph node dissection in 16 patients, chemotherapy in 1 patient, radiotherapy in 1 patient	Reductive chemotherapy in select cases (exophytic tumors)	None specified
Penile Length Changes	N/A	N/A	N/A
Recurrence Rate	17% (8/46 patients)	17.5% at 10 years	12.5% (1/8 patients) at 1 year
Sexual Satisfaction	Some aspects of sexual life, such as manual stimulation/caressing and fellatio, decreased markedly after laser treatment. 6/46 were sexually inactive. 10/46 did not resume sexual activities. 23/46 resumed intercourse. 44/46 did not change masturbation habits.	High; patients judged cosmetic and functional results satisfactory	High satisfaction reported due to preserved function and appearance
Life satisfaction	50% satisfied with whole life, comparable to general population	N/A	N/A
Follow-up Duration	Mean: 4.5 years (Range: 6 months-15 years)	Median: 66 months (Range: 35-132 months)	10 years
Patient Satisfaction with Aesthetic Outcome	High satisfaction with cosmetic results	Excellent cosmetic and functional results reported	Cosmetically excellent results achieved (physicians' opinion)
Penile-Sparing vs. Non-Sparing	Laser therapy preserved function but had recurrence risk	Organ preservation successful with effective local disease control	Effective penile preservation with functional and aesthetic benefits
Treatment Protocol	Laser ablation for tumors <3 cm in size, high or intermediate grade differentiation	Peniscopically controlled laser excision, with adjunctive vaporization of lesion margins	Super-pulsed mode, 5-8 watts; 8-10 mm margin of uninvolved tissue vaporized
Previous Treatments	CO_2 laser therapy for localized penile carcinoma	CO_2 laser excision, peniscopy-guided surgery	CO_2 laser vaporization
Comparison with Other Treatments	Better preservation of function compared to penectomy	Comparable or superior to traditional surgery in terms of functional preservation	Lower relapse rates compared to 5-FU cream and photodynamic therapy
Adverse Effects & Complications	No major complications reported	Minimal complications, no infections reported, minor local edema	No major complications; only one recurrence in the meatal area
Scarring & Discoloration	Minimal	Minimal	No hyperplastic scars

Impact of Cancer Stage	Most effective for early-stage penile carcinoma	Most effective for Tis and T1 lesions	Effective for carcinoma in situ (Erythroplasia of Queyrat)
Penile Sensation	N/A	Preserved in all cases	
Short vs. Long-Term Recovery	N/A	Healing completed in 6 weeks; no need for additional treatment in most cases	Full healing within 14-28 days
Year of Publication	2008	2008	2005
Erectile Function	65% of sexually active men before treatment resumed sexual activity post-treatment with small reduction is satisfaction.	No reported sexual dysfunction post-treatment	No sexual dysfunction reported post-treatment
Body Image Issues	Comparable life satisfaction to general population	High satisfaction with preserved penile form and curvature	High satisfaction with cosmetic results
Limitations	Small sample, retrospective nature, no randomized comparison	Retrospective design, lack of comparison with other conservative treatments	Small sample size, retrospective design
Psychological Counseling Received	No - somewhat less satisfied with their psychological health.	N/A	N/A
Effect Measures Used	Comprehensive 60-Q questionnaire. Carried out by the same physician. Compared to the national average.	N/A	N/A

Supplementary Table S2. Summary of Findings After Nd:YAG Laser Therapy

Category	Schlenker Study (2010)[13]	Tewari Study (2007)[14]	Frimberger Study (2002)[15]
Laser Type	Nd:YAG (neodymium-doped yttrium-aluminium-garnet)	Nd:YAG (Neodymium-doped yttrium-aluminium-garnet)	Nd:YAG
Study Title	Organ-Preserving Nd:YAG Laser Therapy for Penile Carcinoma: A Long-Term Follow-Up	Nd:YAG Laser Treatment of Early-Stage Carcinoma of the Penis Preserves Form and Function of Penis	Penile Carcinoma: Is Nd:YAG Laser Therapy Radical Enough?
Study Design	Retrospective Case Series	Retrospective Study	Retrospective Study
Sample Size	54	32	29 patients (17 with carcinoma in situ, 10 with T1, 2 with T2 cancer)
Age Range/Mean Age	Mean 57.6 years (Range 25-89)	32-67 years, Median: 47 years	Range: 30-93 years, Mean: 55.1 years
Cancer Stage	Tis (11 patients), T1 (39 patients), T2 (4 patients)	pT1 (25 patients), pT2 (7 patients)	Tis (17 patients), T1 (10 patients), T2 (2 patients)
Post-treatment Pain or Discomfort	N/A	No reported need for parenteral analgesics	N/A
Main Findings	Laser therapy offers organ preservation with functional benefits but carries a high recurrence rate	Nd:YAG laser treatment for early-stage penile cancer provides good oncological control while preserving function	Nd:YAG laser therapy is an effective alternative to amputation for early-stage penile carcinoma
Tissue Preservation Success	Good for early-stage cases	100% penile preservation	Complete penile preservation in treated patients
Satisfaction with Urinary Function	N/A	All patients could urinate in standing posture	N/A
Authors	Boris Schlenker, Derya Tilki, Michael Seitz, Markus J. Bader, Oliver Reich, Peter Schneede, Edwin Hungerhuber, Christian G. Stief, Christian Gratzke	Mallika Tewari, Mohan Kumar, Hari S. Shukla	D. Frimberger, E. Hungerhuber, D. Zaak, R. Waidelich, A. Hofstetter, P. Schneede
Additional Treatments	Radical circumcision for additional safety and hygiene	None specified	Groin dissection in 10 of 12 patients with invasive tumors
Libido Changes	N/A	N/A	8/12 patients report regular sexual activity
Overall Survival	One patient died due to recurrent carcinoma	No mortality related to carcinoma	All patients were alive at follow-up
Orgasmic Function	N/A	N/A	excellent
Penile Length Changes	Better preserved than in penectomy	N/A	N/A
Recurrence Rate	42% (16/54 patients), mean time to recurrence: 53 months	6.25% (2/32 patients), occurring at 48-60 months	T1: 1 patient; CIS: 1 Patient
Sexual Satisfaction	Higher compared to amputation	High, with 23 patients reporting normal sexual function. 1 patient information was not available.	All patients satisfied with cosmetic and functional outcomes
Depression/Anxiety Levels	Some patients experienced psychological distress due to recurrence	N/A	N/A
Follow-up Duration	Mean 87 months (Range 9-366 months)	Median 70 months (Range 6-120 months)	Mean: 46.7 months (Range: 6-180 months)
Patient Satisfaction with Aesthetic Outcome	Up to 80% of patients reported satisfactory cosmetic results (Skeppner)	All patients reported satisfaction	all patient satisfaction with cosmetic results
Penile-Sparing vs. Non-Sparing	Better functional preservation but requires long-term monitoring	Preserved penile function while maintaining oncological control	Comparable oncological outcomes with superior cosmetic and functional preservation
Treatment Protocol	Nd:YAG laser coagulation (30-50 W power, 100s, 3mm margin), acetic-acid mapping, circumcision in uncircumcised patients	Local excision with 3-5 mm margin, laser coagulation of tumor bed, circumcision in all cases	Nd:YAG laser coagulation with 3 mm safety margin; acetic acid mapping before treatment
Previous Treatments	Nd:YAG laser coagulation, radical circumcision	Nd:YAG laser excision and in situ coagulation	Nd:YAG laser coagulation
Statistical Significance	No significant difference in recurrence rates between Tis and invasive carcinoma (P = 0.574)	N/A	N/A
Comparison with Other Treatments	Higher recurrence rate than glansectomy but better functional outcomes	Comparable long-term outcomes to partial penectomy with better functional results	Comparable to partial amputation in recurrence rates; better functional and psychological outcomes
Self-esteem	N/A	N/A	N/A

Adverse Effects & Complications	Higher recurrence rates, possible late recurrences (even after 53 months)	Minimal, no wound infections or major complications	No major complications or side effects reported
Scarring & Discoloration	Minimal	Minimal	Minimal
Impact of Cancer Stage	Higher recurrence in advanced stages, not recommended for routine T2 cases	Best suited for early-stage carcinoma (T1/T2)	Effective for Tis and T1; caution in T2 cases
Penile Sensation	Self-reported sensitivity not or only a little impaired	N/A	N/A
Short vs. Long-Term Recovery	Fast recovery but requires long-term follow-up due to late recurrences	Fast healing with epithelialization completed in 7-9 weeks	N/A
Year of Publication	2010	2007	2002
Erectile Function	in line with Skeppner's. Up to 60% of sexually active patients before surgery remained active post-treatment	23 patients reported normal sexual function post-treatment	4/12 had erectile dysfunction/no desire - 8/12 patients report regular sexual activity
Body Image Issues	Better outcomes compared to penectomy	All patients satisfied with cosmetic results	Better psychological outcomes compared to amputation
Limitations	Long-term recurrences, small sample size	Small sample size, lack of randomized trials	Small sample size, retrospective nature, lack of long-term randomized trials
Psychological Counseling Received	N/A	N/A	none of the patients required
Suicidal Thoughts or Distress	N/A	N/A	None reported
Effect Measures	N/A	N/A	N/A

Supplementary Table S3. Summary of Findings After Tm:YAG Laser Therapy

Category	Musi Study (2018)[16]
Laser Type	Tm:YAG (Thulium-yttrium-aluminium-garnet)
Study Title	Thulium-yttrium-aluminium-garnet (Tm:YAG) laser treatment of penile cancer: oncological results, functional outcomes, and quality of life
Study Design	Retrospective Study
Sample Size	26 patients
Age Range/Mean Age	Median: 61 years (Range: 54-72)
Cancer Stage	pTis (47.8%), pT1a (30.4%), pT2 (13.0%), pT3 (8.7%)
Post-treatment Pain or Discomfort	Mild discomfort in external meatus cases
Main Findings	Tm:YAG laser therapy preserves penile structure and function while providing effective oncological control
Time to Sexual Activity Resumption	82.6% resumed intercourse. more than half had erection less than a week. 4 patients had erection after a month. 60% had sexual intercourse within a month. 13% did not continue intercourse.
Tissue Preservation Success	All patients had full penile preservation
Authors	Gennaro Musi, Andrea Russo, Andrea Conti, Francesco A. Mistretta, Ettore Di Trapani, Stefano Luzzago, Roberto Bianchi, Giuseppe Renne, Stefano Ramoni, Matteo Ferro, Deliu Victor Matei, Marco Cusini, Luca Carmignani, Ottavio de Cobelli
Additional Treatments	None specified
Penile Length Changes	78.2% reported no changes in length
Recurrence Rate	17.4% (4/23 patients), including 13.0% invasive recurrence
Sexual Satisfaction	56.5% reported an impact on sexual life; 43.5% reported no change
Follow-up Duration	Median: 24 months (Range: 15-30 months)
Penile-Sparing vs. Non-Sparing	Functional preservation with a similar recurrence rate to other laser modalities
Treatment Protocol	RevoLix 200W continuous-wave laser, 360μm fiber, 15-20W power; safety margin of 3mm vaporized
Previous Treatments	Tm:YAG laser ablation
Comparison with Other Treatments	Comparable recurrence rates to CO_2 and Nd:YAG laser therapy
Adverse Effects & Complications	Minimal post-operative mild edema of the prepuce and mild pain while urinating for a week registered
Scarring & Discoloration	Minimal
Impact of Cancer Stage	Most effective for early-stage (pTis-pT1a); higher recurrence risk in pT2+ cases
Penile Sensation	26.1% maintained sensitivity, 56.5% reported improved sensation, 17.4% worsened
Short vs. Long-Term Recovery	Healing by secondary intention in 5 weeks; no major complications
Year of Publication	2018
Erectile Function	Unaltered post-treatment; 52.2% resumed erections within a week
Limitations	Retrospective study, short follow-up duration
Effect Measures Used	6-Q questionnaire about sexual life

Supplementary Table S4. Summary of Findings of Other Laser Treatments

Category	Windahl Study (2004)	Shaker Study (2023)	Skeppner Study (2015)
Laser Type	Combined CO_2, Nd:YAG	N/A (laser ablation group)	CO_2,
Study Title	Sexual Function and Satisfaction in Men After Laser Treatment for Penile Carcinoma	Saving More Than Just Skin: A Study of Penile Sparing Approaches in Cancer Treatment	Dyadic Aspects of Sexual Well-Being in Men with Laser-Treated Penile Carcinoma
Study Design	Retrospective Study	Retrospective Review	Prospective Observational Study
Sample Size	67 patients (46 participated in interviews)	20 patients	29 patients
Age Range/Mean Age	Mean: 64 years (Range: 34-90)	N/A	Median: 60 years (Range: 37-73)
Cancer Stage	Tis-T2, G1-G2, tumors <3 cm in diameter	T1, T2	pT1 (12 patients), pT2 (14 patients)
Post-treatment Pain or Discomfort	Dyspareunia reported in 10%	No major complications or adverse effects reported	10/21 sexually active men had dyspareunia before treatment, reduced to 2/17 at follow-up
Main Findings	Laser therapy provides high rates of functional and aesthetic satisfaction while preserving sexual function	Laser ablation offers strong local control with better quality of life compared to more invasive procedures	Laser treatment allows organ preservation but can lead to reduced sexual function and satisfaction
Time to Sexual Activity Resumption	Typically, 2-3 months post-treatment	N/A	4/21 patients stopped penetrative sex but have other forms of sexual activity within a year but the rest started intercourse. 8 patients were sexually inactive before treatment which remained unchanged.
Tissue Preservation Success	Full penile preservation in all cases	Penile-sparing success in all cases	Penile preservation achieved in most cases
Authors	T. Windahl, E. Skeppner, S.-O. Andersson, K. S. Fugl-Meyer	Shaker G., Shaker M.	Elisabet Skeppner, Kerstin Fugl-Meyer
Additional Treatments	17 patients had bilateral inguinal lymph node dissection, 1 received adjuvant chemotherapy	None specified	None specified
Libido Changes	Unchanged in 80%, decreased in 17%, increased in 1 patient	N/A	Decreased sexual interest in 45% before treatment, 34% after treatment
Overall Survival	N/A	N/A	N/A
Recurrence Rate	19% (13/67 patients); 10 successfully re-treated with laser	Overall, 5-year recurrence-free survival: 80%; highest in glans excision group (93.8%)	N/A
Partner Satisfaction	N/A	N/A	low sexual desire before and after (8/29 then 9/29). Decreased female lubrication went down (5/29 to 2/29). partner sexual satisfaction remained at around 25 and 24 from 29 participants. factors of life satisfaction either improved slightly or remained unchanged.
Sexual Satisfaction	50% satisfied/very satisfied; 72% considered their sexual life to be as good as they wanted - median 3 years after - (75%) had resumed sexual activity resumed activities	Higher in laser ablation and circumcision groups compared to glans excision	61% satisfied with sexual life before treatment, 32% after treatment ($p=0.039$)
Depression/Anxiety Levels	N/A	N/A	Anxiety in 17% of partners before treatment, 0% at follow-up
Follow-up Duration	Range 6 months-15 years (Median: 3 years)	Mean: 57 months	12 months
Disease-Free Survival	95% disease-specific survival	N/A	N/A
Patient Satisfaction with Aesthetic Outcome	78% satisfied/very satisfied	N/A	N/A
Bias Assessment	N/A	N/A	N/A
Penile-Sparing vs. Non-Sparing	High satisfaction, effective local control with laser therapy	Penile-sparing options reduced impact on sexual function compared to traditional penectomy	Laser treatment preserved function but resulted in some sexual dysfunction
Treatment Protocol	Combined CO_2 and Nd:YAG laser for localized penile carcinoma	Laser ablation for penile cancer; circumcision and glans excision used as comparisons	Organ-sparing CO_2 laser treatment
Previous Treatments	Carbon dioxide (CO_2) and Nd:YAG laser therapy	Laser ablation, circumcision, glans excision	CO_2 laser treatment

Comparison with Other Treatments	Comparable to radiotherapy; better functional outcomes than partial/total amputation	Laser ablation had best sexual function outcomes; glans excision had best local control	Compared to penectomy, laser therapy had better preservation of function
Adverse Effects & Complications	Dyspareunia in 10%, erectile dysfunction in 22%	No major complications or adverse effects reported	among 21 sexually active 2 reported dyspareunia.
Scarring & Discoloration	N/A	N/A	N/A
Impact of Cancer Stage	Most effective for Tis-T2 tumors <3 cm	Effective in T1/T2 penile cancer cases	Most effective for early-stage penile carcinoma
Penile Sensation	N/A	N/A	15/29 patients reported decreased penile sensitivity at follow-up
Short vs. Long-Term Recovery	Full healing typically within 3 months	N/A	N/A
Year of Publication	2004	2023	2015
Erectile Function	Unaltered in 72%, decreased in 22%, improved in 6%	Better preserved in laser ablation and circumcision groups compared to glans excision	IIEF-5 score ≥22 in 14 patients before treatment, 10 patients at 1-year follow-up
Body Image Issues	78% were satisfied/very satisfied with cosmetic results	N/A	N/A
Limitations	Retrospective design, recall bias due to long follow-up period	Small sample size, retrospective nature	Small sample size, single-center study, no control group
Psychological Counseling Received	N/A	N/A	7 patients and 5 partners discussed sexual issues with healthcare professionals
Effect Measures Used	N/A	N/A	IIEF-5, LiSat-11, HADS
method of assessment	N/A	N/A	Hospital Anxiety and Depression Scale, International Index of Erectile Function-5, and Life Satisfaction checklist, LiSat-11. The interviews contained the same questions for patients and partners at all three measuring points.

Supplementary Table S5 - Sexual Function Preservation by Laser type

Laser Type	Study	n/N	% Preserved	Follow-up	Notes
Nd:YAG	Schlenker 2010	32/54	59.30%	Median 87 mo	No validated tool; majority no change in frequency
Nd:YAG	Frimberger 2002	8/12*	66.70%	Mean 46.7 mo	Only sexually active subset (n=12/29)
Nd:YAG	Tewari 2007	23/32	72.00%	Median 70 mo	87% of sexually active men reported no change in satisfaction
**Pooled Nd:YAG**	—	63/98	64.30%	—	—
CO_2	Conejo-Mir 2005	8/8	100%	Up to 10 y	All resumed activity within 6 weeks; no dysfunction
CO_2	Bandieramonte 2008	177/224	79.00%	Median 66 mo	Dyspareunia reduced (10/21→2/17); 4/21 unable to penetrate
**Pooled CO_2**	—	185/232	79.70%	—	—
Combined CO_2 + Nd:YAG	Windahl 2004	33/46	71.70%	Median 3 y	Decline in manual stimulation and fellatio; overall positive
Combined CO_2 + Nd:YAG	Skeppner 2008	30/46	65.20%	Median 3 y	Significant decrease in sexual satisfaction (p=0.039)
Combined CO_2 + Nd:YAG	Skeppner 2015	30/46	65.20%	1 y	Follow-up confirmation; unchanged from baseline
**Pooled Combined**	—	63/92	68.50%	—	—
Tm:YAG	Musi 2017	19/23	82.60%	1 y	Median sexual function score 85/100
**Overall (all laser types)**	—	330/485	68.00%	—	—

Supplementary Table S6 - Cosmetic Outcomes and Body Image Issues

Laser Type	Study	n/N	% Positive Outcome	Outcome Definition	Follow-up	Notes
Nd:YAG	Schlenker 2010 - Satisfaction	43/54	79.60%	Satisfaction with appearance	Median 87 mo	Improved body image assessed separately
Nd:YAG	Schlenker 2010 - Improved body image	38/54	70.40%	Improved body image vs baseline	Median 87 mo	—

Nd:YAG	Frimberger 2002	29/29	100%	Satisfactory/excellent result	Mean 46.7 mo	Rapid healing
Nd:YAG	Tewari 2007	32/32	100%	Satisfactory appearance	Median 70 mo	Full re-epithelialization 3-4 weeks
**Pooled Nd:YAG**	—	142/157	90.40%	Satisfaction or improved appearance	—	Includes satisfaction & body image measures
CO_2	Conejo-Mir 2005	8/8	100%	Excellent appearance	Up to 10 y	Full epithelialization 14-28 days
CO_2	Bandieramonte 2008	—	—	High cosmetic satisfaction	Median 66 mo	Life satisfaction = general population
**Pooled CO_2**	—	—	—	High cosmetic satisfaction	—	One narrative-only study prevents pooled calculation
Combined CO_2 + Nd:YAG	Windahl 2004	36/46	78.30%	Satisfaction with penile appearance	Median 3 y	—
Combined CO_2 + Nd:YAG	Skeppner 2008	36/46	78.30%	Satisfaction with genital appearance	Median 3 y	Same cohort as Windahl
Combined CO_2 + Nd:YAG	Skeppner 2015	36/46	78.30%	Satisfaction with genital appearance	1 y	Follow-up confirmation
**Pooled Combined CO_2 + Nd:YAG**	—	36/46†	78.30%	Satisfaction with genital appearance	—	Based on one unique cohort reported in three studies
Tm:YAG	Musi 2017	18/23	78.30%	No change in penile length	1 y	Surrogate for cosmetic stability

Supplementary Table S7 -Extent of Penile Tissue Preserved by Laser Type and Study

Laser Type	Study	n/N	% Preserved	Outcome Definition	Follow-up	Notes
CO_2	Conejo-Mir 2005	8/8	100%	Penis retained at follow-up	Up to 10 y	—
CO_2	Bandieramonte 2008	215/224	96.00%	Penis retained at follow-up	Median 66 mo	—

**Pooled CO_2**	—	223/232	96.10%	—	—	—
Nd:YAG	Schlenker 2010	54/54	100%	Penis retained at follow-up	Median 87 mo	—
Nd:YAG	Frimberger 2002	29/29	100%	Penis retained at follow-up	Mean 46.7 mo	—
Nd:YAG	Tewari 2007	32/32	100%	Penis retained at follow-up	Median 70 mo	—
**Pooled Nd:YAG**	—	115/115	100%	—	—	—
Combined CO_2 + Nd:YAG	Windahl 2004	46/46	100%	Penis retained at follow-up	Median 3 y	—
Combined CO_2 + Nd:YAG	Skeppner 2008	46/46	100%	Penis retained at follow-up	Median 3 y	Same cohort as Windahl
Combined CO_2 + Nd:YAG	Skeppner 2015	46/46	100%	Penis retained at follow-up	1 y	Same cohort as Windahl
**Pooled Combined CO_2 + Nd:YAG**	—	46/46†	100%	—	—	Based on one unique cohort reported in three studies
Tm:YAG	Musi 2017	26/26	100%	Penis retained at follow-up	1 y	—
Mixed	Shaker 2023 (laser group)	20/20	100%	Penis retained at follow-up	Median 57 mo	—
**Overall (all laser types)**	—	430/439	97.90%	—	—	—

Supplementary Table S8 - Recurrence rates by laser type and study

**Laser Type**	**Study**	**n/N**	**% Recurrence**	Heterogenicity	**Follow-up**	**Notes**

				Outcome Definition		
CO_2	Conejo-Mir 2005	1/8	12.50%	Local recurrence	Up to 10 y	CIS recurrence
CO_2	Bandieramonte 2008	39/224	17.40%	Local recurrence	Median 66 mo	—
**Pooled CO_2**	—	40/232	17.20%	—	—	—
Nd:YAG	Schlenker 2010	23/54	42.60%	Local recurrence	Median 87 mo	Includes higher-stage tumors
Nd:YAG	Frimberger 2002	2/29	6.90%	Local recurrence	Mean 46.7 mo	—
Nd:YAG	Tewari 2007	2/32	6.25%	Local recurrence	Median 70 mo	—
**Pooled Nd:YAG**	—	27/115	23.50%	—	—	—
Combined CO_2 + Nd:YAG	Skeppner 2008	8/46	17.40%	Local recurrence	Median 3 y	Same cohort as Windahl
Combined CO_2 + Nd:YAG	Windahl 2004	—	—	—	Median 3 y	Recurrence data integrated in Skeppner
**Pooled Combined**	—	8/46	17.40%	—	—	Based on one cohort
Tm:YAG	Musi 2017	4/23	17.40%	Recurrence (local/invasive)	1 y	3 invasive (13.0%), 1 non-invasive
**Overall (all laser types)**	—	79/416	19.00%	—	—	—
